# Organized Sports, Overweight, and Physical Fitness in Primary School Children in Germany

**DOI:** 10.1155/2013/935245

**Published:** 2013-02-28

**Authors:** Clemens Drenowatz, Ronald P. Steiner, Susanne Brandstetter, Jochen Klenk, Martin Wabitsch, Jürgen M. Steinacker

**Affiliations:** ^1^Division of Sports and Rehabilitation Medicine, Ulm University Medical Centre, 89075 Ulm, Germany; ^2^Institute of Epidemiology and Preventive Medicine, University of Regensburg, 93053 Regensburg, Germany; ^3^Institute of Epidemiology and Medical Biometrics, Ulm University, 89075 Ulm, Germany; ^4^Division of Pediatric Endocrinology and Diabetology, Ulm University Medical Centre, 89075 Ulm, Germany

## Abstract

Physical inactivity is associated with poor physical fitness and increased body weight. This study examined the relationship between participation in organized sports and overweight as well as physical fitness in primary school children in southern Germany. Height, weight, and various components of physical fitness were measured in 995 children (7.6 ± 0.4 years). Sports participation and confounding variables such as migration background, parental education, parental body weight, and parental sports participation were assessed via parent questionnaire. Multiple logistic regression as well as multivariate analysis of covariance (MANCOVA) was used to determine associations between physical fitness, participation in organized sports, and body weight. Participation in organized sports less than once a week was prevalent in 29.2%, once or twice in 60.2%, and more often in 10.6% of the children. Overweight was found in 12.4% of the children. Children participating in organized sports more than once per week displayed higher physical fitness and were less likely to be overweight (OR  =  0.52, *P* < 0.01). Even though causality cannot be established, the facilitation of participation in organized sports may be a crucial aspect in public health efforts addressing the growing problems associated with overweight and obesity.

## 1. Introduction

In most industrialized countries, including Germany, the prevalence of overweight and obesity in children and adolescents has increased over the last decades [1–3]. This has also led to the occurrence of various metabolic or cardiovascular disease risks, previously only observed in adults [4, 5]. In addition to higher risks for cardiovascular disease and type II diabetes, increased body weight has been associated with psychosocial problems such as depression or low self-esteem [6] and decreased overall quality of life [7]. As excessive body weight tracks into adulthood and increases the risk for CVD, diabetes, or cancer [8, 9], childhood overweight has also been associated with premature mortality [10–12]. Overweight or obesity is a complex phenotype but various risk factors such as low levels of physical activity (PA) and increased sedentary behavior [13], parental obesity and activity levels [14, 15], or socioeconomic status [16] have been identified. 

Along with the change in body composition a secular decline in physical fitness and motor ability in children has been observed [17–19]. The decline was most pronounced for aerobic fitness [20], which is associated with reduced CVD risk [21, 22]. A commonly discussed reason for the decline in physical fitness is low levels of moderate-to-vigorous physical activity [23, 24]. Specifically a decline in habitual physical activity including active transport has been observed [25, 26]. On the other hand participation in organized sports was maintained or even increased [27, 28]. In Germany, 70.2% of 7- to 10-year-old children are involved in sports clubs [29]. This number also reflects the importance of sports clubs concerning social integration and development of the personality [30, 31] in addition to physical fitness and health. Due to its popularity, sports participation should be considered as an effective strategy to reduce fatness and increase fitness in children [32–34]. The World Health Organisation (WHO) also promotes the use of existing settings based on the national situation and cultural habits for the prevention of overweight and obesity [35]. 

While an inverse relationship between body composition and extracurricular sports participation was shown in adolescents [34], no such relationship was shown in primary school children [36]. Quinto Romani [37] concludes that the literature examining the effects of organized sports on children's weight status or physical fitness is inconclusive. In addition to problems of accurate assessment, sports participation in organized youth sports does not necessarily guarantee sufficient physical activity to achieve health benefits or increase fitness [38]. Further, various environmental factors need to be considered when examining the effect of sports participation on body composition and fitness. The purpose of the study, therefore, was to examine the association between participation in organized sports and physical fitness as well as overweight in 8-year-old children while considering various environmental constraints.

## 2. Materials and Methods

### 2.1. Subjects

Baseline data from 1119 (53% male, 47% female) second-grade children (7.6 ± 0.4 years) from 32 schools (64 classes) participating in a health and lifestyle intervention project (URMEL-ICE) in southwest Germany was used. The study protocol was approved by the institutional ethics review board, and parental consent as well as child assent was obtained prior to data collection.

### 2.2. Anthropometry

Height and weight were measured in 1064 children (95% of the total sample) according to standard procedures during a physical examination performed by the outpatient clinic of the Children's hospital. Height was measured to the nearest 0.1 cm (Ulm stadiometer, Busse, Ulm, Germany) and weight to the nearest 0.1 kg using a calibrated balance beam scale (Seca, Hamburg, Germany) with participants wearing only underwear. Body mass index (BMI) was calculated (kg/m²) and converted to BMI percentiles (BMIPCT) using Kromeyer-Hauschild et al. [39] reference values. Overweight was defined as BMIPCT ≥ 90 [39]. 

### 2.3. Physical Fitness

The “Allgemeiner sportmotorischer Test für Kinder (AST)” (general motor abilities test for children) [40], a commonly used fitness test for 6- to 11-year-old children, was administered in the schools by trained personnel. In addition to the 6 items of the AST (6 min run, 20 m sprint, medicine ball throw, throw-on-target, throw-and-turn, and obstacle run) sit-and-reach test and one-leg-balancing from the Eurofit test [41] were incorporated to cover a wider range of fitness-related parameters. A total of 995 children (89% of all participating children) participated in these physical fitness tests.

### 2.4. Sports Participation

 A parental questionnaire was used to determine participation in organized sports outside the school setting. As there is currently no validated instrument for the assessment of health behaviour available in German, questions were based on the KiGGS survey, which assessed health behaviour in 18,000 German children and adolescents [42]. Children's sports participation was classified as less than once a week, once or twice a week, and more than twice a week. 

### 2.5. Confounding Variables

 In addition, the questionnaire assessed confounding variables such as migration background, parental education, parental overweight and parental participation in organized sports. Confounding variables were dichotomized. Migration background was present when either the child's father or mother was born outside Germany or when a foreign language was spoken during the child's first years. Level of parents' education was differentiated by 10 years of schooling of either father or mother. A BMI cutpoint of 25 was used to determine between overweight and nonoverweight, and parental sports participation was separated by participation in organized sports or visiting a fitness centre more than one hour per week. 

### 2.6. Statistical Analysis

Descriptive statistics for the three sports participation groups were calculated and checked for normal distribution. The odds ratio for children being overweight as well as sports participation was calculated via multiple stepwise logistic regression entering sex, overweight, migration background, level of parents' education, parental overweight, and parental participation in sports as confounding variables. In addition, the child's weight status was considered for the sports participation model, while sports participation was considered when calculating the odds ratio for being overweight. Differences in individual fitness parameters by sports participation were calculated via multivariate analysis of covariance (MANCOVA), again adjusting for the previously mentioned confounding variables. Bonferroni adjustment was used for post hoc analyses in case of significant group differences. Missing values were excluded per case, and level of significance was defined as *P* < 0.05. All statistics were calculated in SPSS (17.0.0).

## 3. Results

Table 1 shows age and anthropometric data of the total sample by sports participation. The majority of children (60%) participated in sports once or twice per week beyond the recommended 3 classes of PE. BMIPCT differed significantly between sports participation groups (*F* (2, 1004) = 6.81, *P* < 0.01). Specifically, those reporting sports participation 1-2 times per week had significantly lower BMIPCT than those reporting less than once a week. 

Table 2 displays the prevalence of potential confounding variables on child's sports participation. After entering all of these variables as well as sex in a multiple stepwise logistic regression, comparing children who participate less than once with those participating regularly in organized sports, only migration background as well as parental weight status remained in the final model, which explained between 11.2% (Cox and Snell *R* ^2^) and 16.4% (Nagelkerke *R* ^2^) of the variance in sports participation. Having a migration background or overweight parents reduced the odds for regular sports participation. It was further shown that the odds of children being overweight were significantly lower with increased sports participation (Table 3). 

Using MANCOVA, controlling for sex, overweight, migration background, parental overweight, and parental participation in organized sport, a significant main effect of participation in organized sports on most physical fitness parameters was shown (Table 4). Post hoc analysis using Bonferroni adjustment revealed that children who participate less than once a week in organized sports performed significantly worse than those who participate regularly. There were no differences between children participating once or twice per week in organized sports compared to those participating more often. No significant differences occurred for the 20 m sprint and obstacle run, even though there was a tendency of a better performance with higher sports participation. No association between sports participation and ball aiming was shown.

## 4. Discussion

 In the present study 12.4% of children were overweight or obese, which is comparable to previously reported percentages in Germany and other European countries [43–45]. Similarly, participation rates in organized sports are comparable to those reported previously in German children [29]. The inverse relationship between fitness and overweight as well as physical activity and overweight has generally been accepted [46]. This study, however, further shows that this association remains even after controlling for various family-related confounding variables. These results suggest that sports participation is an important aspect when examining overweight and physical fitness, even at a relatively young age. Regular participation in organized sports, even only once or twice per week, already reduced the odds for being overweight by almost 50%. Similar results have been reported in adolescents [37, 47], 10- to 11-year-old children [48], and in preschoolers [3]. Interestingly, no association between sports participation and body fatness was observed in studies examining similar age groups [36, 49]. One explanation for these results may be that BMI does not necessarily differentiate between body fat and lean tissue. A lower amount of body fat in children with higher sports participation could be masked by a higher lean body mass, which would result in no differences in BMI. Zahner et al. [36], however, relied on skinfold measurements rather than BMI but could not show any association between BMI and sports participation either. These authors argue that engagement in organized sport may not necessarily reflect intensity. Further, only limited information on the type of sports children are engaging in is available, and different sports may affect body composition differently. In addition, sports participation is only one component of children's physical activity, and especially younger children probably obtain the majority of their physical activity via free play [50], which may also differ significantly in intensity. Nevertheless, engagement in organized sports has been associated with long-term engagement in physical activity [51]. The present study also indicates that migration status as well as parental weight status needs to be considered when examining the association between sports participation and body weight in children. Parental overweight as well as lower socio-economic status has been shown to be associated with a higher body weight in children [52]. Migration status is also commonly associated with lower socioeconomic status [53] but a recent study in a German sample showed an inverse relationship between migration status and sports participation independent of household income or parental education [16]. This could be due to different cultural values and attitudes towards sports. Even though migration status is associated with educational level, no significant association between parental education and overweight in children was observed in the present study. Van der Horst et al. [54] also argued that parental support, such as transportation to training and competition, is a crucial component concerning children's sports participation. This kind of support seems to be more important than parents serving as role models, but more active parents are probably more likely to provide transportation or other forms of support for their children [55].

In addition, a significant relationship between sports participation and various components of physical fitness has been shown, independent of migration background, parental education, and parental sports participation. Particularly the role of regular sports participation in aerobic fitness should be emphasized as the inverse association between aerobic fitness and cardiovascular disease risk has already been reported in children [56]. Regular sports participation also increases power, flexibility, and balance [36, 57]. The better performance in these components may be crucial for continued engagement in sports and physical activity, as a better overall fitness allows for better performance and thus increases long-term motivation. Interestingly, Saar and Jürimäe [48] showed such an association between sports participation and fitness only in adolescents, but not in children. In this study, however, only endurance and flexibility were assessed. It should also be considered that younger children generally display lower engagement in organized sports [36] and obtain most of their exercise via unstructured play [50]. Bürgi et al. [58] argue that especially participation in vigorous activity increases physical fitness, but such activities can occur in unstructured play or general physical activity as well. Further, it could be argued that a prolonged engagement in sports is necessary to cause changes in physical fitness, and particularly in younger children, some may have just started participating in organised sports and, therefore, might not display any adjustments.

It should also be considered that the directionality of the relationship between regular sports participation and physical fitness cannot be determined via a cross-sectional study. It may be possible that children with a higher physical fitness are more likely to participate in organized sports due to their better abilities. Similarly, it remains to be determined whether increased body weight is a result of a lack of sports participation or whether selection bias leads to a lower participation of overweight children in organized sports. Most likely the association between sports participation and physical fitness as well as body composition is bidirectional, which will make it difficult to establish a causal pathway even in longitudinal studies. Nevertheless, it has been argued that participation in sports is one way to increase fitness and reduce body fat [33, 59] even though biological and genetic aspects need to be considered.

In addition to the cross-sectional design other limitations of the present study should be addressed. Relying on parent questionnaires for children's participation in organized sports may have introduced potential bias, particularly as parents were informed about the intention of the study due to the following intervention. There is also no information on the intensity children experienced during training sessions or the duration of their training. In general, however, training in organized sports at this age lasts between 60 and 90 minutes per day. Further, only participation in organized sports was considered rather than total physical activity, which may be problematic, especially in younger children. Ebenegger et al. [60], however, did show a direct relationship between sports participation and habitual PA in preschoolers. The large sample size, on the other hand, is a strength of the present study, and an analysis of missing value did not show any systematic bias. In addition, anthropometric as well as physical fitness measurements were performed by trained staff and followed standard procedures, which adds to the credibility of the study.

## 5. Conclusions

 Overall, the present study shows that increased participation in organized sports is associated with increased physical fitness and lower risk for overweight in elementary school children. Even though directionality of these relationships cannot be established, these results emphasize the necessity for an early establishment of an active lifestyle, including sports participation, to allow for the development of various components associated with physical fitness as this may facilitate long-term engagement in sports and physical activity. Considering the low levels of physical education in school with the continued risk of further reduction, the promotion and facilitation of engagement in organized sports would be an important aspect concerning public health. In Germany, roughly 70% of boys and 65% of girls engage in organized sports [29], which could significantly contribute to children's overall physical activity levels as long as sufficient activity time and engagement are assured. In addition, participation in organized sports has been positively associated with the development of psychosocial components such as increased self-esteem, better stress tolerance, and reduced social isolation [61]. These benefits along with the association between sports participation and body weight underline the importance of organized sports in a child's development. Therefore, the facilitation of access to various sports that allow children with different abilities to engage in a variety of activities is a crucial aspect in today's efforts concerning public health.

## Figures and Tables

**Table 1:** Subjects characteristics of the total sample (all) stratified by participation in organized sports per week. Values are mean and SD.

*N* (% male)		Children's participation in organized sports per week		
	All 1053 (53.5%)	Less than once 307 (47.6%)	1-2 times 634 (54.9%)	More often 112 (61.6%)
Age (years)	7.6 (0.5)	7.6 (0.4)	7.5 (0.5)	7.6 (0.4)
Height (cm)	127.1 (5.6)	127.2 (5.5)	127.1 (5.6)	127.2 (6.0)
Weight (kg)	26.4 (5.0)	27.3 (5.6)	26.0 (4.7)	26.2 (4.9)
BMIPCT	49.9 (28.8)	55.1 (30.5)	47.6 (28.1)	49.0 (25.8)

**Table 2:** Distribution of the occurrence of confounding variables of the total sample (all) stratified by participation in organized sports per week. Indicated are *N* and %.

		Children's participation in organized sports per week		
	All *N* (%)	Less than once *N* (%)	1-2 times *N* (%)	More often *N* (%)
Overweight	124 (12.4)	56 (19.5)	61 (10.0)	7 (6.6)
Parental education ≤ 10 y	569 (57.3)	198 (70.7)	314 (52.0)	57 (52.3)
Migration background	259 (26.1)	137 (50.0)	102 (16.7)	20 (18.5)
Maternal overweight	341 (33.4)	131 (44.4)	178 (28.8)	32 (29.4)
Paternal overweight	573 (58.6)	171 (62.4)	338 (56.6)	64 (59.8)
Maternal organized sport	285 (27.5)	40 (13.2)	119 (31.9)	46 (41.8)
Paternal organized sport	272 (27.7)	47 (17.1)	192 (32.4)	32 (29.1)

**Table 3:** Odds ratio (OR) for overweight by participation in organized sports based on logistic regression adjusted for migration and parental weight status.

Participation in organized sports	Overweight (logistic regression)			
	OR	95 % confidence interval		*P*
Once or twice	0.522	0.327	0.834	0.007
More often	0.312	0.126	0.773	0.012

**Table 4:** Mean values and SD for individual fitness tests by participation in organized sports. *F* and *P* values are from MANCOVA.

	Children's participation in organized sports per week			MANCOVA	
	Less than once	1-2 times	More often	*F*	*P*
20 m sprint (s)	4.99 (0.48)	4.83 (0.41)	4.74 (0.42)	2.370	0.092
Sit and reach (cm)	0.56 (6.52)	2.14 (5.95)	3.00 (5.66)	6.841	0.001*
Medicine ball shot put (cm)	277.53 (69.47)	294.17 (63.27)	305.23 (60.57)	5.723	0.003*
6 min endurance run (m)	800.64 (123.16)	866.47 (138.86)	897.37 (124.57)	5.504	0.004*
Obstacle run (s)	25.21 (4.49)	23.71 (4.57)	22.96 (4.07)	2.555	0.078
Throw-and-turn (points)	13.43 (10.46)	16.58 (11.25)	19.57 (11.52)	4.120	0.017*
Ball aiming (points)	7.75 (3.78)	8.21 (4.19)	9.23 (4.31)	0.928	0.396
One leg balancing (points)	1.02 (0.57)	0.84 (0.57)	0.81 (0.62)	3.585	0.028*

*Difference between less than once a week and weekly sports participation.
